# Pediatric pre-hospital emergencies in a rural region in northern Germany: frequency, urgency, patterns of utilization, and comparison with adult cases

**DOI:** 10.1186/s12873-026-01653-1

**Published:** 2026-06-27

**Authors:** Janina Dombrowski, Ulrike Stentzel, Heiko Krause, Mona Ebermann, Christian Oehlke, Lutz Fischer, Berthold Henkel, Timm Laslo, Wolfgang Hoffmann, Bibiana Metelmann, Olaf Schoffer, Neeltje van den Berg

**Affiliations:** 1 Section Epidemiology of Health Care and Community Health, Institute for Community Medicine, University Medicine Greifswald, Greifswald, Germany; 2 German Center for Child and Adolescent Health (DZKJ), partner site Greifswald/Rostock, Greifswald, Germany; 3 Public Emergency Medical Services, District of Vorpommern-Greifswald, Greifswald, Germany; 4 Department of Anaesthesiology, Intensive Care, Emergency and Pain Medicine, University Medicine Greifswald, Greifswald, Germany; 5 Central Emergency Department, University Medicine Greifswald, Greifswald, Germany; 6 Center for Evidence-Based Healthcare, Faculty of Medicine, University Hospital Carl Gustav Carus, TUD Dresden University of Technology, Dresden, Germany

**Keywords:** Secondary data analysis, Emergency medical service, Ground rescue, Pre-hospital, Rural, Low-density population areas, Pediatric, Child, Age-specific differences

## Abstract

**Background:**

Pediatric emergencies are rather rare in pre-hospital care. The aim of this study was to analyze the frequency and urgency of pediatric emergency medical service (EMS) missions and to identify age-specific characteristics in EMS utilization in the rural district of Vorpommern-Greifswald in northeastern Germany.

**Methods:**

In this retrospective observational study, all emergency protocols from ambulance vehicles and emergency physician vehicles from January 1, 2022, to December 31, 2023, in the study region were analyzed using descriptive statistical methods. Patients were categorized into age groups: infants (< 1 year), toddlers (1–5 years), schoolchildren (6–13 years), adolescents (14–17 years), adults (18–64 years), and seniors (≥ 65 years).

**Results:**

Over the study period, 71,446 patients had an EMS contact, thereof 255 infants (0.4%), 976 toddlers (1.4%), 1,437 schoolchildren (2.0%), 1,395 adolescents (2.0%), 25,220 adults (35.3%), and 42,163 seniors (59.0%). The annual emergency rate among adults ≥ 18 years was nearly three times higher than among minors < 18 years (169 vs. 58 patients per 1,000 inhabitants within the respective age groups). Pediatric cases accounted for 5.7% of all missions. Vehicle dispatches for minors followed a bimodal temporal distribution with peaks between 12:00 and 1:00 p.m. and between 6:00 and 7:00 p.m. Seasonal variation was observed, with the highest dispatch volume in June (10.5% of all pediatric dispatches) and the lowest in February (5.3%). Trauma was more common in minors compared to adults (41.8% vs. 23.1%), with the highest proportion among schoolchildren (52.2%). The proportion of critically ill or injured patients (NACA IV–VII) was lower in minors than in adults (10.2% vs. 20.8%). Physician involvement was more frequent in pediatric missions than in adult missions (39.4% vs. 32.9%). Infants showed the highest physician involvement (53.5%).

**Conclusions:**

In pediatric missions, the involvement of physicians is high, however, critically ill children are rare. Low exposure to pediatric cases may contribute to insecurity among paramedics and emergency physicians in pediatric emergencies. Therefore, hands-on training in pediatric emergency care is essential. Given the rising demand for EMS, telemedicine could be a valuable addition in rural settings to reduce the burden on physician resources and ensure comprehensive pediatric care.

**Clinical trial number:**

Not applicable

## Introduction

Pediatric **e**mergency **m**edical **s**ervice (EMS) missions in Germany are rare, accounting for approximately 5% of all ground-based emergencies [1, 2] and 8–12% of all air rescue missions [3, 4] in Germany. According to the NACA classification for patient’s severity (NACA: National Advisory Committee for Aeronautics), 15–24% of children encountered in EMS missions present with potentially life-threatening conditions (NACA IV–VII) [2–4]. Experience with critically ill or severely injured children are limited [5], and invasive, life-saving interventions are infrequently performed [6]. As a result, EMS personnel lack the opportunity to establish routine competence in pediatric emergency care [7].

Young children often struggle to articulate symptoms, may be uncooperative, and cannot remain still for extended periods [8–10]. This complicates preclinical diagnostics and the assessment of severity. Specific symptoms as well as anatomical and physiological differences between children and adults further necessitate an adapted therapeutic approach, including weight-based medication dosing, airway management, and vascular access [11]. As a consequence, pediatric emergency equipment differs from that used in adults [10].

The lack of routine, combined with these specific challenges, can lead to uncertainty, insecurity and stress among EMS personnel [1]. In a survey, the EMS personnel rated their subjective competence in pediatric emergencies as low [12], with insecurity increasing in younger children [13]. Stress may result in psychological strain [8, 14] and increase the likelihood of medical errors [14]. Pediatric patients are particularly vulnerable to medication errors, as drug dosages are often weight-dependent [15].

Healthcare services are often concentrated in urban areas. In the rural district of Vorpommern-Greifswald, many municipalities lack both outpatient and inpatient medical services, resulting in long travel distances to medical facilities for large parts of the population, especially for children.

The impact of prolonged travel distances on pre-hospital EMS utilization and demands for local EMS providers remains unknown. To the best of our knowledge, no data have been published on pediatric EMS utilization in comparable rural regions. This study investigates pediatric EMS missions in the rural district of Vorpommern-Greifswald, with the aim of analyzing the current care situation in emergency services and identifying age-specific differences in EMS mission frequency, medical urgency, and operational characteristics.

## Methods

### Study area

The rural district of Vorpommern-Greifswald, located in the federal state of Mecklenburg-Western Pomerania in northern Germany, borders the Baltic Sea to the north and Poland to the east. With 237,231 inhabitants spread across 3,927 km² (2023), the district is one of the most sparsely populated regions in Germany (population density approximately 60 inhabitants/km²) [16]. Mecklenburg-Western Pomerania has the highest number of tourist overnight stays per capita among all German federal states, with 19,506 overnight stays per 1,000 inhabitants in 2022; the national average was 5,343 overnight stays per 1,000 inhabitants [17].

In Germany, outpatient care is structured into two independent sectors: the EMS and the association of statutory health insurance physicians (KV). The KV is a self-governing organization of physicians and psychotherapists in Germany who provide outpatient medical care for patients covered by the statutory health insurances. Their responsibilities include ensuring adequate coverage by general practitioners in Germany and organizing the medical on-call service outside regular practice opening hours (nights, weekends, and public holidays). The medical on-call service is intended for lower-acuity cases that cannot wait for the next scheduled physician appointment, while the EMS is designated for time-critical, potentially life-threatening emergencies and the non-emergency medical transport of patients. There is no centralized patient steering mechanism; patients self-select which service to contact.

### Infrastructure of EMS

In Germany, EMS organization falls under federal state legislation, leading to regional differences in the use of emergency vehicles, vehicle staffing, and legal response times. In the federal state of Mecklenburg-Western Pomerania, ground-based EMS is the responsibility of each administrative district.

In the district of Vorpommern-Greifswald, EMS operations are managed by a publicly operated EMS organization in accordance with the regional legal framework. During the study period, the ground-based EMS system comprised 25 rescue stations across the district, where the emergency vehicles were located. A total of 29 ambulance vehicles and 12 emergency physician vehicles were in operation. Dispatching of emergency resources is coordinated centrally by the integrated dispatch center located in the district capital of Greifswald. Experienced paramedics trained in emergency medical dispatch allocate resources based on standardized decision-support protocols and predefined dispatch criteria. The ambulance vehicles (emergency ambulances) are staffed with paramedics. For improved availability of medical expertise, emergency physicians are dispatched separately via emergency physician vehicles to meet the ambulance crew on scene (rendezvous system). The ground-based EMS system is supplemented by tele-emergency physicians - emergency physicians who can be contacted by emergency ambulances at any time through a secure audio–video connection and who are stationed at a central location within the district. During the study period, six of the 29 ambulance vehicles were equipped with devices for contacting tele-emergency physicians.

Air rescue constitutes an important component of preclinical emergency medical care, particularly in rural regions. In the district of Vorpommern-Greifswald, there is one emergency helicopter station in the district capital of Greifswald. Dispatch of emergency helicopters is coordinated by the same integrated dispatch center.

### Classification of patient urgency

The NACA score is an internationally established and widely used instrument that classifies the severity of a patient’s medical condition on an eight-point scale, ranging from no injury or illness (NACA 0) to death (NACA VII).

The “Graded EMS Care System“ (German: Gestuftes Versorgungssystem; *GVS*), originally developed by the EMS system of the city of Cologne [18], is a regionally applied classification system developed for the German EMS context. In contrast to the NACA score, the GVS incorporates not only the patient’s condition but also external circumstances - including weather conditions and the location of the incident - and aims to reflect the appropriate level of required resources and target response time for a given mission. The GVS classification comprises seven levels, each corresponding to a defined level of required resources and response urgency (Table 1). By definition, patients in GVS levels 1–3 are not immediately life-threateningly ill or injured and are thus considered non-urgent cases. Patients assigned to levels 4–7 are classified as urgent, as life-threatening conditions are either present or cannot be ruled out. For missions involving patients in GVS 1–3, the dispatch of a fully equipped ambulance vehicle is considered disproportionate to the level of clinical need (‘overqualified’) [19].

In the district of Vorpommern-Greifswald, operational data are routinely documented during the EMS missions using the standardized electronic reporting system *docYou* (pulsation IT, Berlin). In addition to the mandatory Minimal Dataset in German Emergency Medicine (MIND) [20], which contains the NACA score, EMS personnel have classified patients‘ urgency using the GVS since late 2019. Both classifications were applied independently by the crew of each responding vehicle.

**Table 1:** Assessment of patient’s urgency according to the GVS, adapted from Lechleuthner et al. [18]

GVS level	Patient’s medical condition	Appropriate emergency resource
GVS 1	no emergency, no medical urgency - patient stable	outpatient care
GVS 2	mild disease	non-emergency transport vehicle
GVS 3	acute case - patient stable	medical on-call service of the statutory health insurance system
GVS 4	life-threatening condition cannot be ruled out - patient stable	emergency ambulance
GVS 5	acute life-threatening condition - patient unstable	emergency ambulance + emergency physician vehicle
GVS 6	resuscitation	emergency ambulance + emergency physician vehicle
GVS 7	death (patient already deceased)	physician on-site

### Study design and statistical analysis

In this retrospective analysis of secondary data, all emergency protocols from emergency ambulances and emergency physician vehicles from January 1, 2022, to December 31, 2023, in the rural district of Vorpommern-Greifswald were analyzed. The emergency protocols were anonymized. All primary and secondary missions (interhospital transfers and non-emergency patient transports) conducted by the ground-based EMS were included in the analysis. Emergency protocols from air rescue services were unavailable and therefore not included in the analysis.

The mission protocols were analyzed using descriptive statistical methods. Due to the exploratory nature of the study, descriptive comparisons between age groups were performed without inferential statistical testing. Regional variation in pediatric mission numbers were visualized using cartographic representations.

Patients were grouped into the following age groups: infants (< 1 year), toddlers (1–5 years), schoolchildren (6–13 years), adolescents (14–17 years), adults (18–64 years), and seniors (≥ 65 years). In addition, patients younger than 18 years were summarized as minors and compared to adults ≥ 18 years.

Categorical variables are presented as absolute and relative frequencies. Age is reported as median with interquartile range (IQR). Incomplete records were excluded from the analyses (available case analysis).

All statistical analyses were performed using R (version 4.3.3). Cartographic representations were created with ArcGIS Pro (version 3.5.4, Esri, Redlands, CA: Environmental Systems Research Institute).

### Data processing

An EMS mission can involve multiple patients (e.g., in the case of a traffic accident) and dispatch of several responding vehicles (units). Each unit generates a separate record for every involved patient, which may lead to multiple entries for one patient. As no patient-identifying information were available (anonymized data), records referring to the same patient could not be merged. To avoid bias from duplicate entries, analyses were conducted on three different levels: patient level (demographic and medical characteristics, GVS classification), unit level (date and time of dispatch, mission location, hospital transport), and mission level (mission type, involvement of an on-scene or tele-emergency physician). Separate datasets were created for each level of analysis, with one row corresponding to a patient, a unit, or a mission, respectively.

**Fig. 1:**
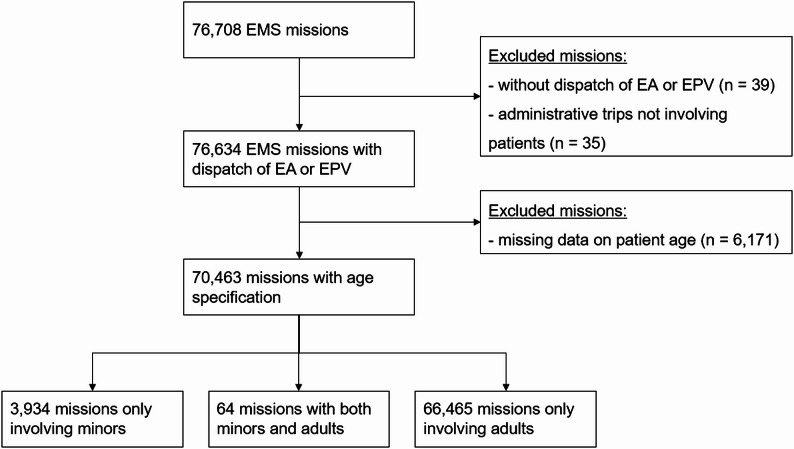
Flowchart of data processing for the mission-level analysis. EA: emergency ambulance; EMS: emergency medical service; EPV: emergency physician vehicle

Figure 1 illustrates the data processing workflow for the mission-level dataset. On the patient and unit levels, contacts between patients and units were analyzed. Duplicate entries on patient level were identified based on mission ID, age and sex. For duplicates at the patient level, the emergency physician’s documentation was used for the analyses when available; otherwise, emergency ambulance records were used. For analyses at the unit level, multiple entries of one unit in a given mission were identified based on mission ID, unit ID, and time of dispatch, and the most complete entry was used in the analysis.

Diagnoses were documented in a standardized way using pre-specified fields from a drop-down menu. If a diagnosis was recorded, it was retrospectively categorized according to the affected organ system: traumata/injuries, diseases of the respiratory system, diseases of the nervous system, cardiovascular diseases, abdominal diseases, psychiatric disorders, other consequences of external causes (e.g., intoxication or anaphylaxis), and other emergencies.

The transport rate to a hospital was calculated as the number of units dispatched in primary missions where the transport destination was a hospital, divided by the total number of units dispatched in primary missions.

The annual rate of emergency patients was calculated by dividing the number of patients within the two-year study period (restricted to missions located in Vorpommern-Greifswald) by two, then normalizing for population size per 1,000 inhabitants in the district of Vorpommern-Greifswald. Population figures as of December 31, 2023, were provided from the State Office for Internal Administration of Mecklenburg-Western Pomerania.

The State Office for Internal Administration of Mecklenburg-Western Pomerania provided data on tourist overnight stays for 2022 and 2023.

## Results

From January 1, 2022, to December 31, 2023, a total of 76,708 EMS missions were documented. After data cleaning, 3,998 missions involving minors and 66,529 missions involving adults were included in the analysis (Fig. 1).

Table 2 summarizes the number of patients, mission frequency, and dispatched EMS units over the two-year study period, stratified by age groups. As missions may involve multiple patients of different ages, and EMS units may treat more than one patient per mission, the overall numbers of missions and units for minors (< 18 years) and adults (≥ 18 years) do not exactly match the sum of individual age categories. Due to incomplete records, case numbers reported in subsequent analyses may differ slightly from Table 2.

**Table 2:** Number of patients, missions, and unit dispatches during the study period from January 1, 2022 to December 31, 2023, stratified by age group. Primary and secondary missions are included

	Patients, *n*	Missions, *n*	Vehicle dispatches, *n*		
			Total	Ambulance vehicles	Physician vehicles
Infants (< 1 year)	255	251	333	240	93
Toddlers (1–5 years)	976	960	1,294	923	371
Schoolchildren (6–13 years)	1,437	1,425	1,786	1,369	417
Adolescents (14–17 years)	1,395	1,373	1,777	1,338	439
**Total minors (< 18 years)**	**4**,**063**	**3**,**998** *	**5**,**188** *	**3**,**869** *	**1**,**319** *
Adults (18–64 years)	25,220	24,818	31,786	24,317	7,469
Seniors (≥ 65 years)	42,163	41,799	51,282	40,942	10,340
**Total adults (≥ 18 years)**	**67**,**383**	**66**,**529** *	**83**,**049** *	**65**,**250** *	**17**,**799** *

* The overall numbers may deviate from the sum of the respective age groups, as in some missions multiple patients were involved and/or a single unit provided care for more than one patient

### Distribution of age

During the study period, 255 infants (0.4%), 976 toddlers (1.4%), 1,437 schoolchildren (2.0%), 1,395 adolescents (2.0%), 25,220 adults aged < 65 years (35.3%), and 42,163 seniors aged ≥ 65 years (59.0%) had an EMS contact (Table 2). The median age of the patients was 69 years (IQR: 52–82 years).

Relative to the respective population, the annual rate of emergency patients in Vorpommern-Greifswald was nearly three times higher among adults than among minors (169 vs. 58 patients per 1,000 inhabitants per year).

**Fig. 2:**
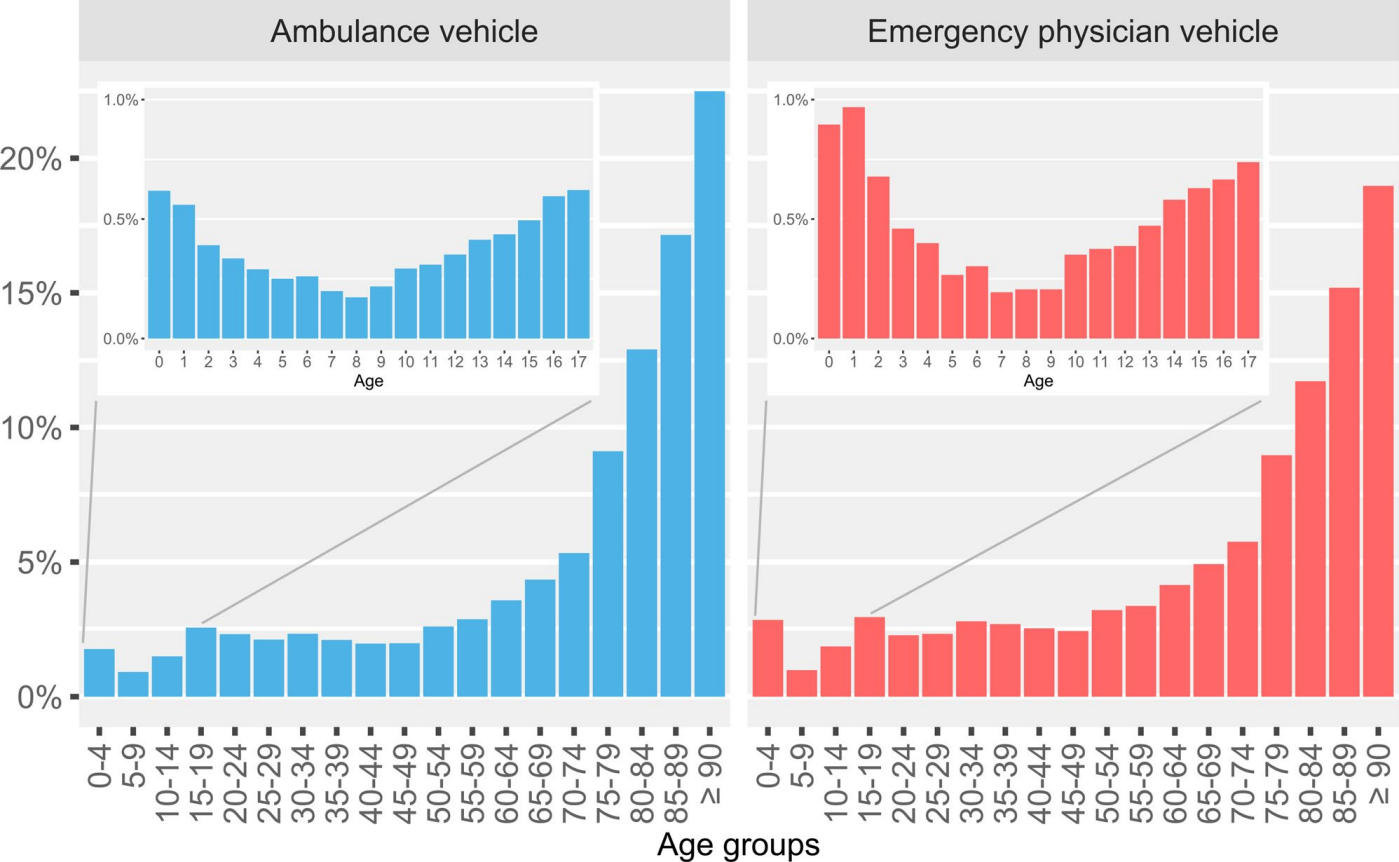
Percentage distribution of unit dispatches in the district of Vorpommern-Greifswald during the study period from January 1, 2022 to December 31, 2023, stratified by age group. To improve interpretability, raw dispatch numbers were adjusted for the population size of each age group. The distribution of ambulance vehicles (*n* = 69,119) and emergency physician vehicles (*n* = 19,118) are presented separately

With increasing age, unit dispatch frequency rose exponentially (Fig. 2). Compared to ambulance vehicles, emergency physician vehicles were relatively more often dispatched to patients under 5 years of age (ambulance vehicle: 1.8% vs. physician vehicle: 2.9%). In contrast, ambulance vehicles were more frequently dispatched to patients aged ≥ 80 years (ambulance vehicle: 52.5% vs. physician vehicle: 45.9%).

### Frequency of pediatric emergencies

Across the two-year study period, pediatric missions accounted for 5.7% of all EMS missions (*n* = 3,998), corresponding to an average of 5–6 pediatric missions per day. The proportion of pediatric missions varied between municipalities in the district of Vorpommern-Greifswald, ranging from 0% to 32%, with a mean of 6.0% ± 4.6% and a median of 5.5% (IQR: 3.2–7.1%).

**Fig. 3:**
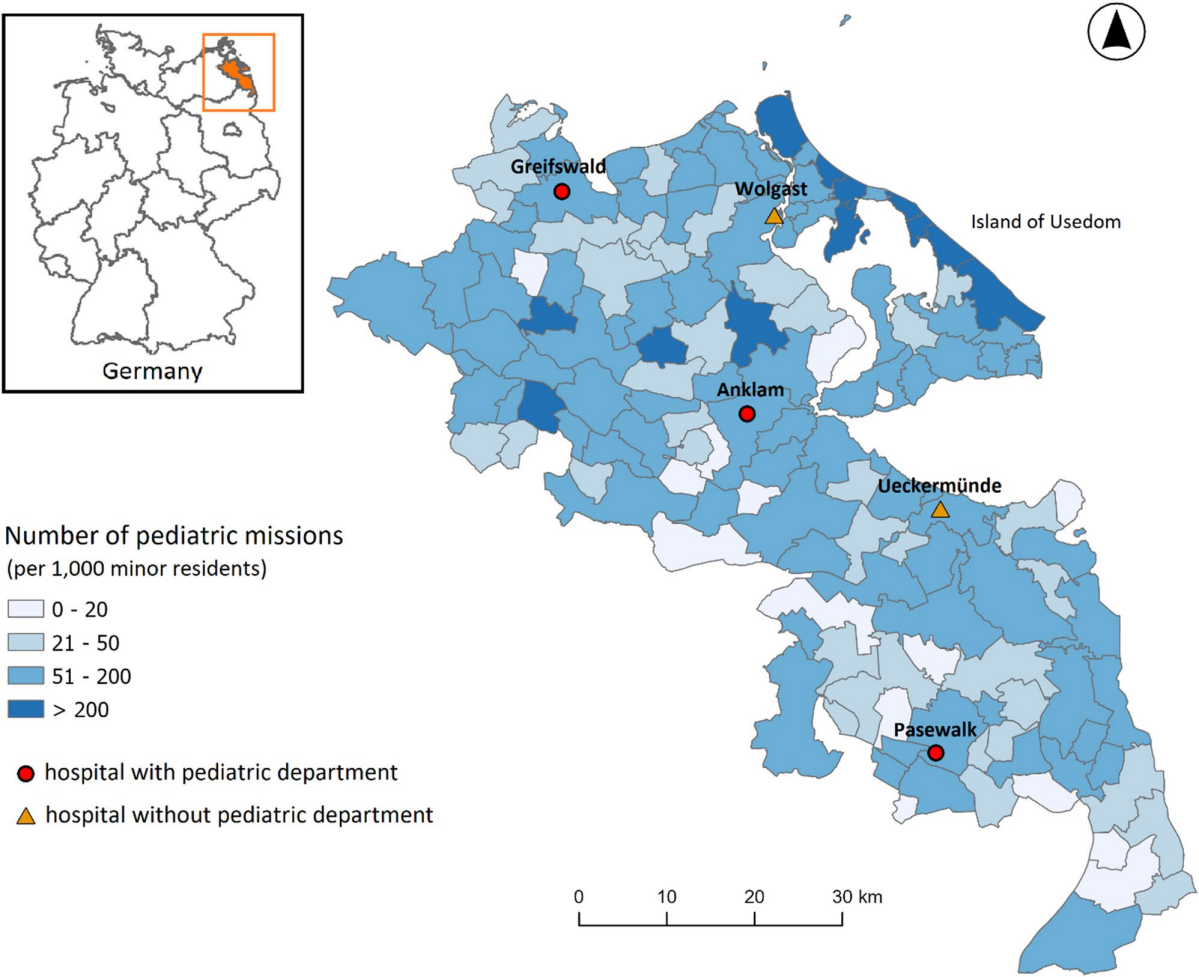
Regional distribution of pediatric missions per 1,000 minor residents in the municipalities of the district of Vorpommern-Greifswald during the two-year study period from January 1, 2022 to December 31, 2023 (*n* = 3,853). In another 145 (3.6%) pediatric missions, no valid mission location was documented or the mission took place outside the rescue service area of Vorpommern-Greifswald (support operations in other districts or federal states of Germany)

Figure 3 shows regional differences in the number of pediatric missions per 1,000 resident children and adolescents. The highest rates were observed in municipalities on the island of Usedom, with up to 477 pediatric missions per 1,000 pediatric residents in the two-year study period. There are nine municipalities with zero pediatric missions during the entire study period.

Among the 4.063 pediatric patients, schoolchildren (35.4%) and adolescents (34.3%) represented the largest groups, followed by toddlers (24.0%) and infants (6.3%). The proportion of male patients under 18 years was 52.9%.

### Time distribution of emergency vehicle dispatches in pediatric missions

**Fig. 4:**
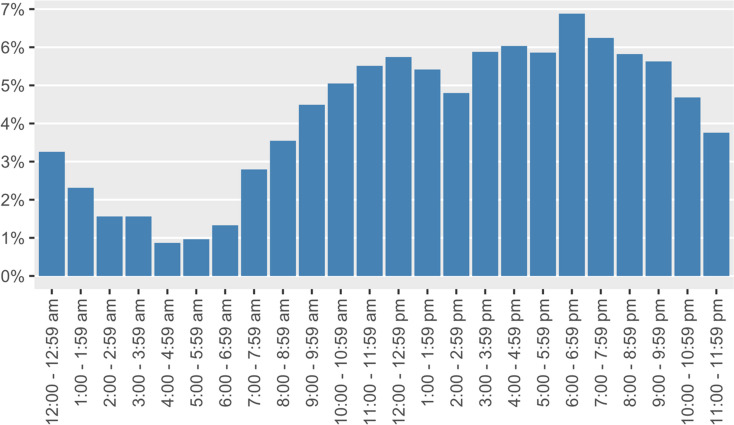
Unit dispatches for pediatric missions by time of day during the study period from January 1, 2022 to December 31, 2023 (*n* = 5,188)

**Fig. 5:**
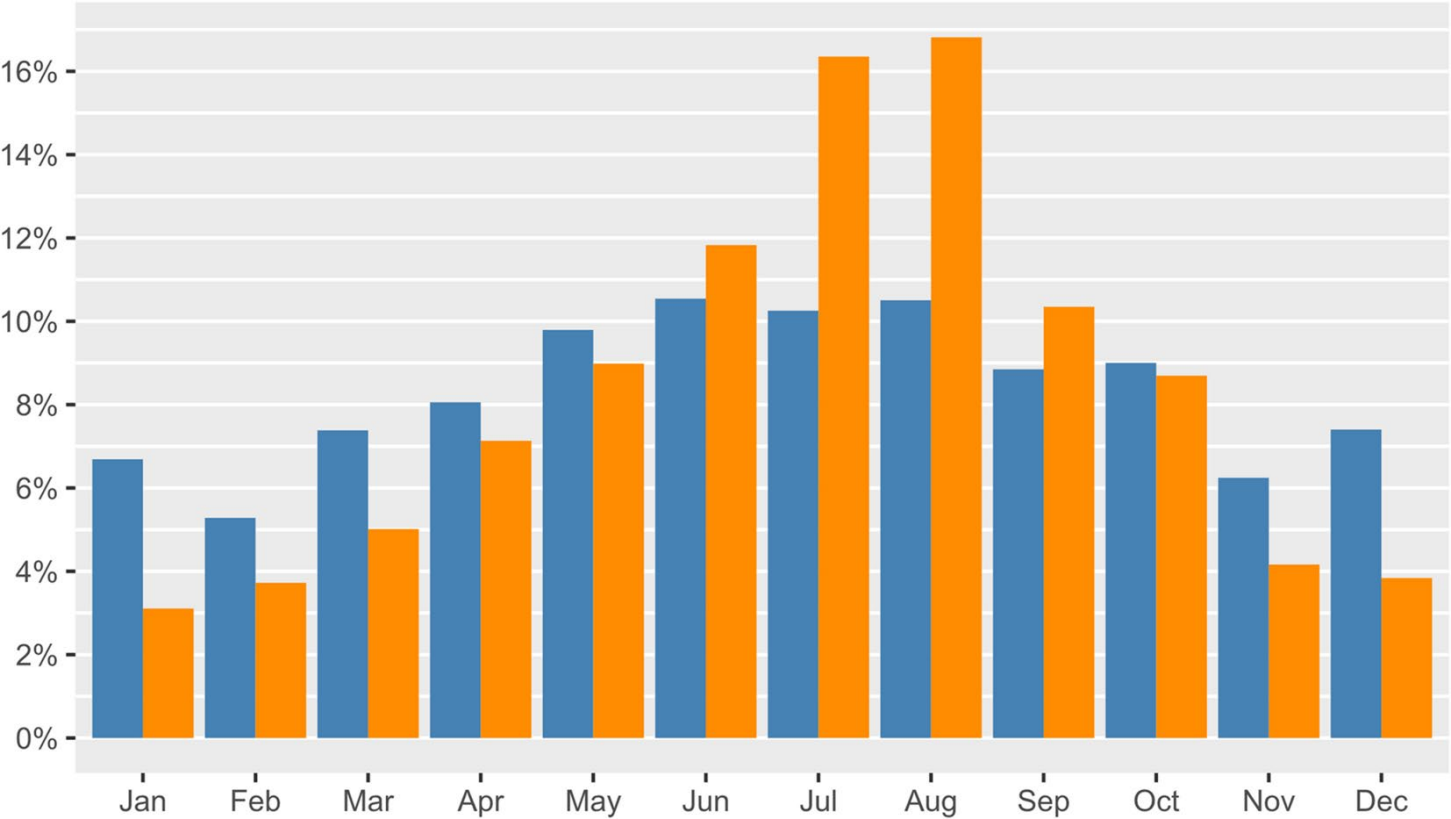
Unit dispatches for pediatric missions (blue, *n* = 5,188) and tourist overnight stays (orange, *n* = 13,061,808) by month in the district of Vorpommern-Greifswald from January 1, 2022 to December 31, 2023

EMS unit dispatches for pediatric missions exhibited a bimodal diurnal distribution with peaks between 12:00 p.m. and 1:00 p.m. and between 6:00 p.m. and 7:00 p.m. (Fig. 4). The lowest activity occurred between 2:00 a.m. and 7:00 a.m. Dispatches were distributed relatively evenly across weekdays (Monday: 14.0%, Tuesday: 13.6%, Wednesday: 14.4%, Thursday: 15.4%, Friday: 15.1%, Saturday: 13.1%, Sunday: 14.3%). Seasonal variation was observed (Fig. 5), with the highest mission volumes in summer (June: 10.5%, July: 10.3%, August: 10.5%) and the lowest in February (5.3%) and November (6.2%).

### Primary missions

Primary missions accounted for 92.9% (*n* = 3,715) of all pediatric EMS missions, compared to 87.5% (*n* = 58,231) among adult missions.

The median transport time (scene-to-hospital) across all primary EMS missions in the district of Vorpommern-Greifswald was 18.4 min. Substantial regional variation was observed: median transport time at the municipality level ranged from 5.3 to 47.3 min, with the longest transport time recorded for a municipality on the island of Usedom.

**Fig. 6:**
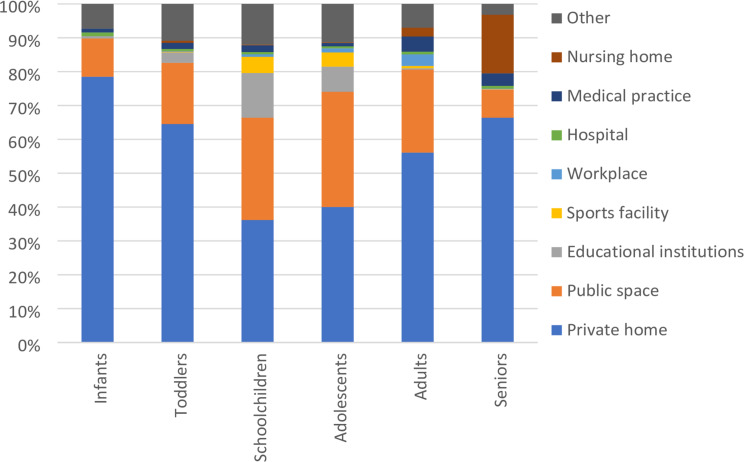
Location of primary missions by age group during the study period from January 1, 2022 to December 31, 2023: infants (< 1 year, *n* = 275 unit dispatches), toddlers (1–5 years, *n* = 1,216), schoolchildren (6–13 years, *n* = 1,673), adolescents (14–17 years, *n* = 1,688), adults (18–64 years, *n* = 29,170), and seniors (≥ 65 years, *n* = 44,867)

Across all age groups, the majority of primary missions occurred in private homes (Fig. 6), with a total of 47.0% for pediatric patients. This proportion was lower among schoolchildren (36.2%) and adolescents (40.0%), who more frequently required EMS in public spaces (30.2% and 34.0%) or educational institutions (13.2% and 7.4%). Among seniors, 83.7% of missions occurred in the residential setting, including 66.4% in private homes and 17.3% in nursing homes.

In total, for 52,716 (83.9%) patients the working diagnosis could be categorized and analyzed (among infants: 136 [67.3%], toddlers: 720 [78.5%], schoolchildren: 1,151 [85.8%], adolescents: 1,156 [87.6%], adults: 19,379 [84.7%], seniors: 30,174 [83.5%]). In 1,678 cases (2.7%), it was documented that no disease or injury was identified (infants: 15 [7.4%], toddlers: 30 [3.3%], schoolchildren: 35 [2.7%], adolescents: 28 [2.1%], adults: 582 [2.5%], seniors: 988 [2.7%]). No diagnosis was assigned in the remaining patients.

**Fig. 7:**
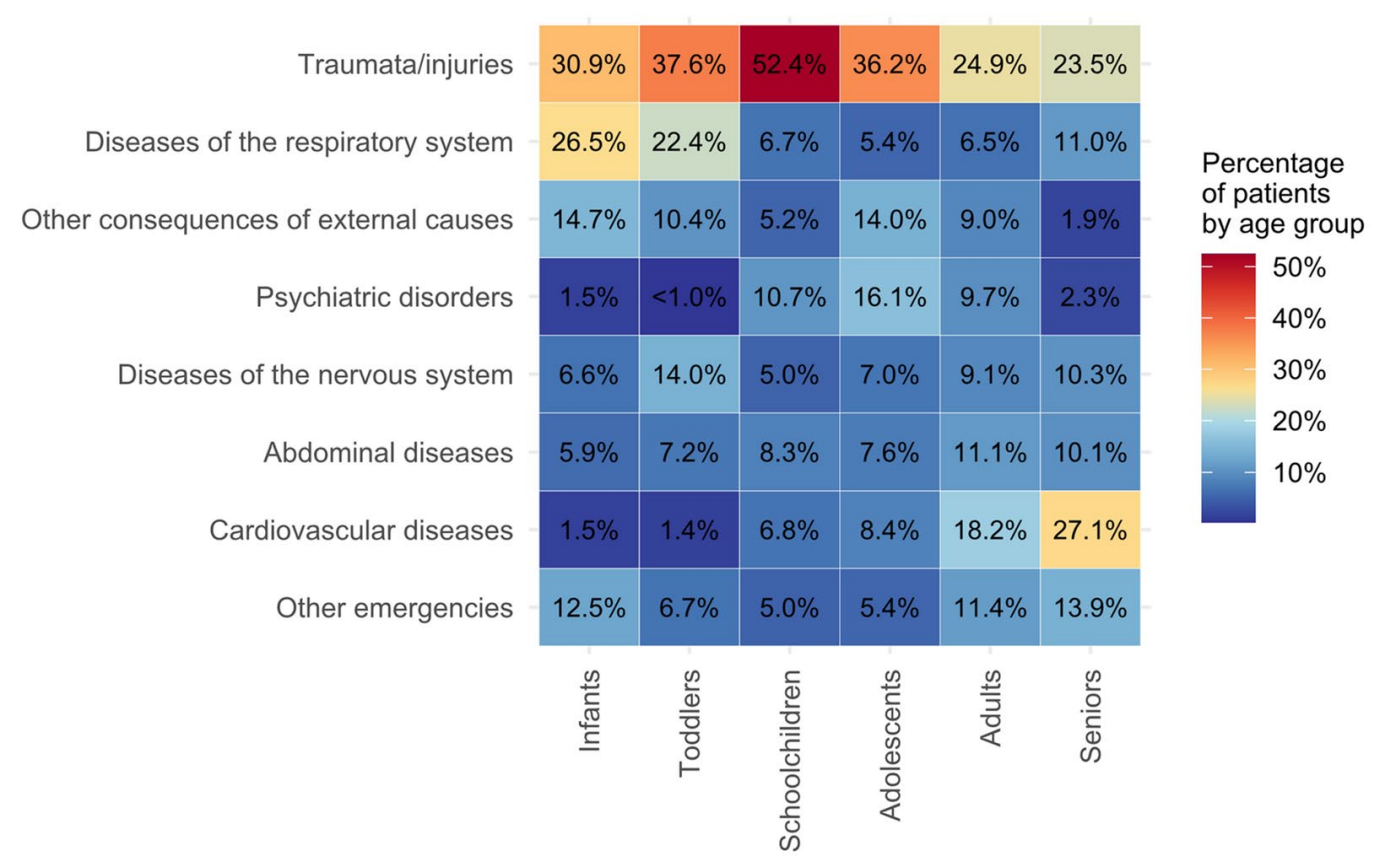
Diagnosis categories of patients in primary missions, stratified by age group during the study period from January 1, 2022 to December 31, 2023: infants (< 1 year, *n* = 136 patients), toddlers (1–5 years, *n* = 720), schoolchildren (6–13 years, *n* = 1,151), adolescents (14–17 years, *n* = 1,156), adults (18–64 years, *n* = 19,379), and seniors (≥ 65 years, *n* = 30,174). Percentages are displayed as column percentages

The distribution of diagnosis categories differed by age group (Fig. 7). Overall, traumata/injuries accounted for the majority of diagnoses, with the highest proportion among schoolchildren (52.4%). Traumata/injuries were more frequent among minors than adults ≥ 18 years (42.2% vs. 24.0%). In both groups, the majority of injuries involved the extremities (minors: 44.6% vs. adults: 42.2%) and craniofacial regions (minors: 39.4% vs. adults: 31.5%).

In infants and toddlers, respiratory diseases represented an additional large share of diagnoses (26.5% and 22.4%). Cardiovascular diseases were mainly recorded in adults (18.2%) and seniors (27.1%).

The category *other consequences of external causes* accounted for 9–15% of diagnoses in infants, toddlers, adolescents, and adults. Among adolescents and adults, this category mainly included intoxications (88.9% (*n* = 144) and 76.1% (*n* = 1,331), respectively). In adolescents, these intoxications were primarily due to alcohol (59.0%), medications (16.7%), illicit drugs (16.0%), and other unspecified substances (6.3%). In infants, most of the diagnoses in this category were aspirations (*n* = 9), burns/scalds (*n* = 5), and in toddlers, mainly anaphylaxis (*n* = 28, 37.3%), burns/scalds (*n* = 15, 20.0%), and intoxications (*n* = 18, 24.0%).

Psychiatric disorders accounted for 9–17% of diagnoses among schoolchildren, adolescents, and adults. Suicidality was documented in no infants or toddlers, but in 15 (1.3%) schoolchildren, 41 (3.5%) adolescents, 371 (1.9%) adults, and 56 (0.2%) seniors.

The distribution of the “Graded EMS Care System“ (GVS) for primary missions with respect to pediatric patients was as follows: 21.0% GVS 1 (n = 793), 17.4% GVS 2 (n = 659), 30.9% GVS 3 (n = 1,166), 28.2% GVS 4 (n = 1,064), 2.4% GVS 5 (n = 92), 0.1% GVS 6 (n = 4), and < 0.1% GVS 7 (n = 1). The proportion of urgent cases (defined as GVS 4–7) was lower among minors compared to adults (30.7% vs. 45.1%). Similarly, the proportion of life-threatening ill or injured patients (NACA IV–VII) was lower in minors than in adults (10.2% vs. 20.8%). Among minors, infants exhibited the highest rate of life-threatening conditions (NACA IV–VII: 14.2%).

During the two-year study period, a tele-emergency physician was involved in 1,128 (1.8%) primary missions, mainly for adults (*n* = 1,097). Total physician involvement including on-scene and tele-emergency physicians in pediatric primary missions was higher than in adult missions (39.4% vs. 32.9%). 77.5% (*n* = 2,790) of the 3,601 emergency ambulances dispatched for pediatric primary missions transported a patient to a hospital. In adult primary missions, 47,087 (82.6%) transports to a hospital were performed.

**Fig. 8:**
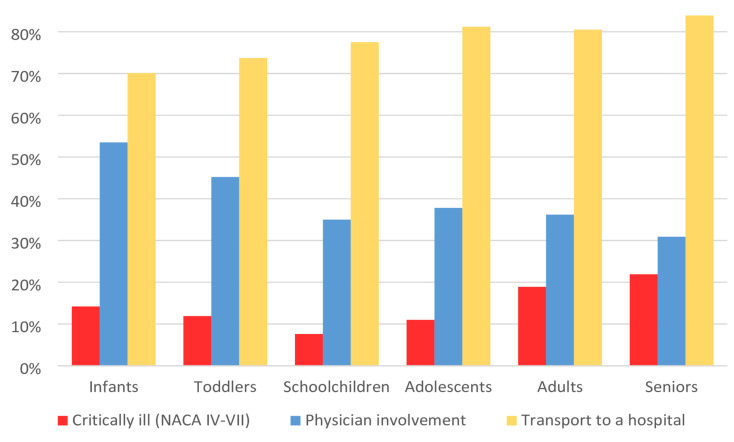
Characteristics of primary missions by age group during the study period from January 1, 2022 to December 31, 2023: infants (< 1 year), toddlers (1–5 years), schoolchildren (6–13 years), adolescents (14–17 years), adults (18–64 years), and seniors (≥ 65 years). The proportion of critically ill patients (NACA IV–VII, *n* = 61,968 patients, red), the proportion of total physician involvement including on-scene and tele-emergency physicians (*n* = 62,040 missions, blue), and the transport rate to a hospital (*n* = 60,620 ambulance vehicles, yellow) are displayed. The bars do not sum up to 100% per age group, as the characteristics may overlap

Infants demonstrated the highest rate of physician involvement, while the proportion of critically ill cases (NACA IV–VII) remained lower than in adults and seniors (Fig. 8). Physician involvement decreased with increasing patient age, whereas the rate of transports to a hospital increased.

## Discussion

In the rural district of Vorpommern-Greifswald, an average of five to six pediatric EMS missions occurred per day during the study period. In these missions, regional, diurnal, and seasonal patterns are observed. In our study, an age-dependent increase in EMS mission numbers was observed, confirming previous findings from the German federal state of Bavaria [21].

Pediatric missions accounted for 5.7% of all EMS missions, consistent with other ground-based EMS systems in Germany [2, 3, 22]. Urban regions in Germany show similar proportions [4, 23]. Pediatric emergencies remain rare. Children under 6 years account for only 1.8% of all missions. In this age group, anatomical and physiological differences compared with adults are most pronounced, necessitating specialized care [10, 11]. The low frequency of pediatric missions limits the ability of EMS personnel to acquire routine experience, contributing to uncertainty among both paramedics and emergency physicians [12, 13]. Regular, practical training in pediatric emergencies is therefore essential [5].

We would recommend simulation-based training targeting high-acuity, low-occurrence (HALO) pediatric scenarios as particularly relevant for rural EMS personnel who encounter these cases infrequently [24, 25]. Concrete examples of such scenarios may include pediatric cardiac arrest and resuscitation, supraventricular tachycardia, severe respiratory failure, anaphylaxis, seizure management, and neonatal emergencies - which are core components of the Pediatric Advanced Life Support curriculum [26]. We recommend regular re-certification intervals to maintain competency in HALO scenarios.

Critically ill pediatric cases are uncommon. In the present study, only 10.2% of children were classified as NACA IV–VII, indicating that 9 out of 10 children had no life-threatening conditions. By comparison, this proportion in adults was twice as high (20.8%). Other studies report higher rates of critically ill children (15–24%) [2–4, 23], however, these analyses focused exclusively on physician-staffed missions, which are more likely to involve life-threatening cases compared to our data set. The finding that children are generally less severely ill or injured than adults [2, 3], can be confirmed by the present study.

The structural context of pediatric primary care in the study region warrants consideration as a potential determinant of EMS utilization. In the district of Vorpommern-Greifswald, pediatric outpatient care is provided by ten practices serving the entire district, the majority of which are concentrated in the district capital of Greifswald. Pediatric inpatient care is available at three hospitals with dedicated pediatric departments within the district. This geographic concentration of pediatric services leaves large parts of the district with limited local coverage. Evidence from Mecklenburg-Western Pomerania suggests that children residing more than 20 km from the nearest pediatrician have fewer physician contacts per year compared to those in better-served areas (32% vs. 50% with four or more annual visits), and are more likely to be seen by non-pediatrician general practitioners when care is sought [27]. While outpatient practices in Germany are required to offer open office hours for acute presentations without prior appointment, practical accessibility in rural areas with limited physician density may be constrained by capacity limitations and extended in-practice waiting times. Taken together, limited access to outpatient pediatric care may plausibly contribute to EMS utilization for non-urgent conditions in the study region, though this hypothesis could not be tested within the scope of the present study.

The absence of a hospital on the island of Usedom (Fig. 3), combined with the generally low hospital infrastructure in the rural district of Vorpommern-Greifswald, poses a challenge to the EMS system. On the island of Usedom, all patients requiring hospitalization must be transported to the mainland, resulting in prolonged travel times. This challenge is even more pronounced for children and adolescents because hospitals with pediatric departments are scarce (Fig. 3). The imminent closure of key healthcare facilities in rural areas can further strain both pre-hospital and hospital emergency providers, as demonstrated in [28]. Due to prolonged transport distances, air rescue constitutes an important component of preclinical emergency medical care in rural regions.

The highest pediatric mission rates per 1,000 minor residents were observed on the island of Usedom. Tourism may partly explain this finding. Usedom is the primary holiday destination in the district of Vorpommern-Greifswald, hosting over five million overnight stays per year [29], predominantly during the summer months. Seasonal peaks in EMS demand may reflect temporary population growth due to tourism. Seasonal peaks challenge EMS systems, as they must be taken into account when planning EMS infrastructure and staffing. In addition, parental uncertainty may contribute to increased EMS utilization, as caregivers who are unfamiliar with local healthcare structures may adopt a more cautious approach concerning their child’s medical condition. Situations that might otherwise be managed without EMS involvement may therefore result in emergency calls while on vacation. It is also conceivable that the high number of pediatric missions on the island of Usedom is partly attributable to the limited hospital infrastructure, which may result in patients contacting EMS rather than accessing hospital care.

Despite the low proportion of critically ill pediatric cases, physician involvement was disproportionately high, especially for infants. In Germany, there is a predefined catalog listing medical conditions that require the presence of an emergency physician [30]. This catalog could partly explain this finding. Given workforce shortages and long travel distances in the rural district of Vorpommern-Greifswald, telemedicine has proven to be a valuable supplement to ground-based EMS for adult patients [31, 32]. In the district of Vorpommern-Greifswald, most telemedicine-supported missions currently involve adult patients, as no tele-emergency physicians with pediatric specialization are available. A pediatric tele-emergency physician could support paramedics and emergency physicians in therapeutic decision-making and might bridge the interval until the on-scene physician arrives.

However, important limitations must be acknowledged. In all pediatric missions in which intravenous medication administration or airway management may be required, tele-emergency consultation alone is not sufficient and cannot replace physical presence. Furthermore, the limited ability to perform a comprehensive remote physical examination is of particular concern in pediatric patients. Key clinical indicators - including capillary refill time, fontanelle tension in infants, peripheral pulse quality, and skin turgor - cannot be adequately assessed via video consultation, yet are central to the pre-hospital evaluation of critically ill children. Regarding technological feasibility, the basic infrastructure for telemedicine in the pre-hospital setting has been operational in the district of Vorpommern-Greifswald since October 2017 [31]. Nevertheless, connectivity problems remain a relevant limitation, particularly in areas with restricted mobile network coverage such as forested or geographically remote locations.

Given the low daily volume of pediatric missions in the district of Vorpommern-Greifswald (approximately 5–6 per day), a dedicated district-level pediatric tele-emergency physician may not be operationally or economically justified. Instead, a state-wide pediatric tele-emergency physician model covering peak hours (Monday to Sunday, 10:00 a.m. to 10:00 p.m.) could support the entire federal state of Mecklenburg-Western Pomerania, leveraging synergies across multiple districts and cities. However, the appropriateness and effectiveness of tele-emergency consultation in pediatric EMS missions has not yet been formally evaluated, and prospective studies are needed before firm implementation recommendations can be made.

Although only 10% of minors were critically ill, nearly 78% of ambulance vehicles involved in pediatric missions transported the pediatric patient to a hospital, indicating that many non-life-threatening cases were transported. Without follow-up data from hospitals, the medical necessity of these transports remains unclear. However, this high transport rate may reflect precautionary decision-making under conditions of diagnostic uncertainty and absence of available alternatives. Implementation of pediatric home visit services offered by the KV or a dedicated tele-medical alternative, may reduce transport rate for low-acuity emergencies.

Previous studies report higher pediatric hospital transport rates (87.4% [3] and 93.1% [33]), potentially due to inclusion of air rescue missions. Consistent with our findings, infants are least frequently transported [3].

Similar to a previous study [2], traumata/injuries were more common among minors than adults, with a slightly higher proportion of injuries among minors than previously reported (42.2% vs. 32.4–36.8% [2–4]). Intoxications were an important cause of EMS missions in adolescents, primarily due to alcohol.

EMS diagnoses should be interpreted as preliminary working hypotheses. In a study conducted in the middle of Germany, nearly 10% of EMS diagnoses differed from emergency department diagnoses [34]. A study conducted in Türkiye obtained similar results for pediatric missions [35]. In the district of Vorpommern-Greifswald, the concordance between pre-hospital and hospital discharge diagnoses remains unknown. A structured comparison of diagnoses implemented as a feedback system could enhance diagnostic accuracy and reliability. In a recent survey conducted in Germany, 95.4% of EMS personnel expressed the need for structured feedback on hospital diagnoses and patient outcomes [36], which could improve long-term treatment quality and patient safety [37]. Due to data protection regulations, German EMS personnel receive little or no feedback on their working diagnoses, and such feedback is predominantly provided through informal channels [36].

Pediatric missions include a higher proportion of non-urgent cases compared with adults (GVS 1–3, 69.3% vs. 54.9%). This suggests that many missions might be managed with alternative, needs-based resources rather than emergency ambulance vehicles [19]. Alternative resources need to be developed and implemented within the EMS system, as there is currently no option other than dispatching an ambulance vehicle for less urgent emergency calls in the rural district of Vorpommern-Greifswald.

### Strengths and limitations

As this study was designed as a descriptive epidemiological analysis, no multivariable regression analyses were performed and reported associations should not be interpreted as causal. Multivariable analyses of EMS mission characteristics should be addressed in future research.

This study is based on secondary data, thereby reflecting the real operational context (real world) along with all the advantages and disadvantages associated with this type of data.

The level of detail in the dataset allowed for the identification of regional variations in the frequency of pediatric EMS missions, which were visualized using geospatial mapping.

A major limitation of this study is the absence of outcome data and the lack of linkage to downstream healthcare utilization. Due to the absence of integrated data infrastructure between pre-hospital EMS documentation systems and hospital information systems in the study region, longitudinal patient tracking across sectoral boundaries was not feasible. Consequently, it remains unclear whether the high transport rate reflects appropriate clinical caution or systematic overtriage, and whether physician involvement was clinically justified in individual cases. Addressing this gap through prospective data linkage across EMS records, hospital information systems, and statutory health insurance data should be a priority for future research.

The absence of integrated data pathways between pre-hospital and hospital care is not merely a methodological limitation of the present study but a broader structural deficit in the German healthcare system with direct implications for patient safety and quality assurance. A recently published German expert consensus position paper on digital emergency management identified the digital integration of all actors along the emergency care chain - from dispatch centers through EMS crews to receiving hospitals - as the central prerequisite for a future-proof emergency medical system [38]. This position paper emphasizes that the goal of digital emergency management is to enable all parties involved to electronically exchange information, access all data relevant to optimal patient care, and communicate seamlessly during emergency interventions. Despite this normative consensus, cross-sectoral data integration remains largely unrealized in the German healthcare system.

The vast majority of studies in the field of pre-hospital emergency care have focused exclusively on emergency physician protocols. By taking into account both the protocols from emergency ambulances and emergency physician vehicles, it was possible to quantify the physician involvement.

To our knowledge, this is the first analysis of ground-based pediatric EMS missions in German rural areas. Cross-state comparisons are challenging due to the federal organization of EMS in Germany, which results in structural differences as well as varying documentation systems [39]. Additionally, the district of Vorpommern-Greifswald is unique due to low population density, demographic structure, and seasonal tourist influx, which may limit generalizability to other regions. While the high number of tourists affects the calculation of mission rates per residents, it represents an essential characteristic of the study region and reflects the actual service demand faced by local EMS providers.

Figure 8 illustrates the proportion of critically ill patients, physician involvement, and transport rates of ambulance vehicles. These parameters were analyzed at the patient, mission, or unit level. Linking them at the individual case level was not possible because of anonymization. The results regarding the relationship between these parameters should therefore be interpreted cautiously.

For the NACA score, the emergency physician’s documentation was used for analysis when available. It can be assumed that emergency physicians are able to assess patient severity more accurately than paramedics. In missions without physician involvement, the documentation of the ambulance personnel was used instead. While this approach may have improved the overall accuracy of NACA score analysis, the NACA rater differed depending on whether or not an emergency physician was involved, which may have influenced the observed association between physician involvement and patient severity.

Two classification systems were used to assess mission severity: while the NACA score provides a clinically focused patient severity index that facilitates comparison with international studies, the GVS captures the broader operational context of each mission and thus reflects the dispatch and resource-allocation logic of the regional EMS system. The parallel use of both instruments allows for both internal operational analysis and external scientific comparability.

A further limitation concerns the validity and reliability of the GVS classification. In contrast to the NACA score, published validation data for the GVS are currently lacking. The developer of the GVS has acknowledged that the instrument may be subject to uncertainty in individual cases [19]. While the developer points to a relatively stable distribution of GVS levels over time as indirect evidence of consistency [19], formal psychometric validation remains absent. Dedicated validation studies are warranted to evaluate the psychometric properties of the GVS, particularly in pediatric patients.

Comparisons of ground and air-based EMS missions in Germany revealed systematic differences in patient populations. Compared to ground-based EMS, air rescue services exhibit a higher proportion of pediatric missions, and patients are more frequently severely ill or injured [3]. For our analysis, only ground-based EMS mission protocols were available and could be included in the analysis, potentially underestimating pediatric mission frequency and critical illness in the rural district of Vorpommern-Greifswald. Therefore, subsequent analyses will focus on air rescue missions.

## Conclusion

German EMS encounters with critically ill children and adolescents are comparatively rare, therefore, there may be uncertainty in the treatment of pediatric patients. The future role of emergency physicians may increasingly focus on pediatric emergencies, complex medical conditions, severe trauma, and highly invasive interventions, which should be reflected in medical education and EMS training.

The absence of integrated data pathways between pre-hospital and hospital care represents a critical systemic deficiency in the German healthcare system. Structured feedback mechanisms between EMS and receiving hospitals would not only serve quality assurance purposes but also enable longitudinal patient tracking and outcome assessment across sectoral boundaries. The implementation of such cross-sectoral data infrastructures should be prioritized to improve the quality of pre-hospital care.

A pediatric tele-emergency physician may support paramedics and emergency physicians and might bridge the interval until the on-scene physician arrives. Given workforce shortages and the high involvement of emergency physicians in pediatric missions, telemedicine may represent a potential complement to ground-based EMS for pediatric patients, though prospective evaluation is needed before firm recommendations can be made.

Seasonal tourist influx and changes in the availability of outpatient and inpatient medical services, especially for pediatric care, should be closely monitored and considered when planning EMS infrastructure in rural regions.

## Data Availability

The datasets used/or analyzed in the present study are available from the corresponding author on reasonable request.

## Abbreviations

EMS: Emergency medical service
GVS: Graded EMS Care System
IQR: Interquartile range
KV: Association of statutory health insurance physicians
MIND: Minimal Dataset in German Emergency Medicine
NACA: National Advisory Committee for Aeronautics
